# Seed germination dynamics of some woody legumes: implication for restoration of arid zones ecosystems

**DOI:** 10.5114/bta.2023.132774

**Published:** 2023-12-21

**Authors:** Toma Buba, Abalis Gaya Ezra, Sunday Paul Bako, Mohammad Umar Sabo

**Affiliations:** 1 Department of Biological Sciences, Faculty of Science, Abubakar Tafawa Balewa University, Bauchi, Nigeria; 2 Department of Biological Science, Ahmadu Bello University, Zaria, Kaduna State, Nigeria; 3 Department of Crop Production, Faculty Agriculture, Abubakar Tafawa Balewa University, Bauchi, Nigeria

**Keywords:** acacia, arid, germination, inertia, seeds, viability

## Abstract

The seed germination dynamics of *Acacia nilotica*, *Bauhinia rufescens*, *Faidherbia albida*, and *Piliostigma reticulatum* were investigated over 28 days. Seeds were pretreated with concentrated sulfuric acid. Determined germination parameters included germination energy, germination period, germination capacity, germination inertia, and viability loss. Seeds exposed to sulfuric acid for extended periods (30, 40, 50, and 60 min) exhibited a higher germination rate (α = 0.05). For *A. nilotica* seeds, the 50 min acid treatment resulted in the highest germination energy of 85.5% and germination capacity of 89.5% (*P* = 0.001); conversely, the 60-min treatment yielded the highest germination energy and capacity, both 96.5% (*P* = 0.079), for *P. reticulatum*. In the case of *B. rufescens*, the 30-min treatment led to the highest germination energy of 93% and germination capacity of 88% (*P* = 0.001). For *F. albida*, all acid treatments resulted in 100% for both germination energy and germination capacity (*P* = 0.621). Viability losses for *A. nilotica* and *P. reticulatum* were higher (32 and 30%, respectively) than those for *B. rufescens* and *F. albida*, which were 19.5 and 6%, respectively (*P* = 0.000). Generally, higher germination energy resulted in lower viability loss, dependent on the species. Analyses of germination inertia and viability loss suggest that seeds of *A. nilotica* and *P. reticulatum* possess a greater ability to survive in arid land climates than those of *B. rufescens* and *F. albida*. However, due to the advantage of lower viability loss, *B. rufescens* and *F. albida* should be preferred for the natural restoration of arid land ecosystems where seed availability is a major concern.

## Introduction

Arid lands are characterized by extreme climatic variability, including high temperatures and erratic seasonal rainfall, in terms of onset, amount, distribution, and duration of the season (Leech, 2013; Marigi et al., 2016). Precipitation in these regions is markedly low relative to the demand required for the proper functioning of the ecosystem (McHugh et al., 2015). The limited moisture availability and extremely high temperatures serve as the most pivotal factors that control bio-logical processes and, therefore, determine productivity in dry lands (Leech, 2013; McHugh et al., 2015). Biotic factors, such as species richness, diversity, abundance, and interactions, are also believed to play key functions in productivity, nutrient cycling, and modulating the effects of other abiotic factors on ecosystem functioning (Colemana and Whitman, 2005). Other abiotic factors, such as geomorphology and edaphic features, impact the redistribution of rainwater and determine the ecosystem structure and functioning at a local level (Maestre, 2016). These factors render arid land ecosystems extremely fragile, and anthropogenic influences, even minor deviations from the mean climate condition, can cause substantial changes in composition, productivity, or the normal functioning of the systems. The ecosystem, therefore, possesses a reduced ability to buffer the effects of climatic extremes and human overexploitation with its scant resources (Malagnoux et al., 2007; Marigi et al., 2016). Both anthropogenic and climatic changes generally result in a negative impact on the ecosystem and the livelihoods of millions of people inhabiting the arid lands; and these problems are expected to exacerbate due to global climatic changes (Leech, 2013; Maestre, 2016).

Leguminous trees play a crucial role under the harsh environmental conditions of arid regions (Amerin and Daldoum, 2017; Tchatchoua et al., 2019). They contribute to maintaining suitable conditions for the normal functioning of the ecosystem and safeguard human enterprises, such as arable cultivation, animal husbandry, and livelihoods (Malagnoux et al., 2007). With a high tolerance to aridity, capabilities of fixing nitrogen, and providing food, microhabitats, and shelter to various species of different organisms, these trees play a key role in the restoration of degraded arid land ecosystems (Fox et al., 2012; Smýkal et al., 2014). The trees are suitable for agroforestry because they do not compete with arable crops for light, nutrients, or water, and also serve as windbreaks, boundary markers, and sources of domestic and industrial raw materials, such as fuelwood, timber, fiber, gum Arabic, and medicinal products, etc. (Chuyong and Acidri, 2015; Akpalu et al., 2019; Yousif et al., 2020).

Trees adeptly utilize the soil and climatic features of arid lands to complete their life cycles (Kuelková et al., 2022). In general, trees initiate their life cycles with seed production, which is followed by successful germination, seedling survival, growth and maturity, and ultimately, the production of new seeds (Mordecai, 2012). Seed survival and the maintenance of their viability are critically important for trees growing amidst the extreme spatial and temporal environmental variables of arid lands (Mordecai, 2012; Lai et al., 2016). To enhance survival in such unpredictable climatic conditions, they employ seed dormancy to synchronize germination under optimal conditions, which are best for the survival of their seedlings, or to spread the germination of batches of seeds over a prolonged period to avoid catastrophic loss of seedlings due to drought, predation, or disease. Leguminous tree species are widely recognized for their mechanical dormancy, a condition whereby germination is restricted due to a hard seed coat enclosing the endosperm and embryo (Nasr et al., 2013; Duncan et al., 2019). The hard seed coat inhibits water imbibition by the seed and therefore slows the germination process. Consequently, the death of seeds that may occur during the germination process due to drought will affect only a few seeds that absorbed water, while most of the seeds that resisted water absorption retain their viability and germinate during the next recruitment opportunities when water is available (Yousif et al., 2020). The degree of mechanical dormancy can vary between seeds of different tree species, among seeds from different individuals of the same tree species, or even among individual seeds from the same tree. Dormancy can also vary among seeds from different individual trees of the same species due to their microhabitat variations, or the varied stages of maturity among the seeds from the same individual tree (Dalling et al., 2011; Opoku et al., 2018).

Arid lands are presently grappling with ongoing deforestation and desertification, and vegetation restoration is viewed as the most effective method to counteract desertification and restore ecosystem integrity. Utilizing native plants in the restoration of degraded arid lands is a principal strategy employed because these plants are highly adaptable to their harsh environment. However, the success of establishing tree plantations is significantly dependent on the ability to secure a large number of seedlings that germinate simultaneously (Lai et al., 2016). Moreover, seed survival and successful germination are important factors through which trees respond to environmental fluctuations in arid lands, driving essential ecosystem processes such as plant population dynamics, community species composition, productivity, and ecosystem services. These processes result in positive feedback loops that ensure the ongoing existence of the ecosystem and the well-being of human populations that depend on it (Mordecai, 2012).

Given the importance of seed survival and germination dynamics to arid land ecosystems, understanding variability in seed survival across different tree species in time and space is critically vital. Unfortunately, such studies, which carry tremendous ecological applications, are often overlooked in ecological studies (Mordecai, 2012). Additionally, afforestation programs aimed at restoration and conservation in arid lands using native tree species are seriously challenged by poor seed germination due to the complication of seed dormancy (Erickson et al., 2016; Kildisheva et al., 2020; Hernandez et al., 2020). To navigate this challenge, rapid and synchronous germination is required. Seed dormancy must be artificially broken to achieve swift, synchronous germination, wherein a large number of uniformly sized seedlings can be obtained (Amerin and Daldoum, 2017; Opoku et al., 2018). This is typically done artificially by subjecting seeds to physical or chemical pre-germination treatments, which soften the hard seed coat and permit water imbibition by the endosperm and embryo (Mordecai, 2012; Yousif et al., 2020). Common pretreatments utilized to overcome seed coat dormancy include soaking in concentrated acid, hot water, and mechanical scarification (Jibo et al., 2018; Akpalu et al., 2019).

Tree and shrub species found to be suitable for the rehabilitation of dry lands include *Acacia nilotica*, *Faidherbia albida*, *Bauhinia rufescens*, and *Piliostigma reticulatum*. *A. nilotica* is valued for its rapid biological nitrogen-fixing and soil stabilization capabilities. Adapting to extremely erratic climate conditions in the dunes of arid and semiarid lands, it can thrive amid high temperatures, severe drought, and nitrogen-deficient soils (Yousif and Wang, 2021). *A. nilotica* also has significant impacts on enhancing and improving soil fertility and crop yield (Amadou et al., 2020), making it suited for planting on marginal lands. These trees are nitrogenfixing and exhibit strong drought resistance (Toppo, 2020). *B. rufescens* has also proven to be well-adapted to arid areas and possesses the ability to restore degraded lands (Soumana et al., 2016). Numerous researchers have reported that the *F. albida* tree provides multiple ecosystem services to degraded or marginal lands, improving soil fertility through nitrogen fixing, recycling deep horizon soil nutrients, and reducing soil erosion (Lawan et al., 2021). *F. albida* positively impacts soil fertility, microclimate, and the yields of crops that grow beneath its crown. Notably, it sheds its leaves at the onset of the rainy season. These features position the tree as an excellent agroforestry species, enhancing soil fertility and organic matter, as well as the yields and nutritional values of crops (Tougiani et al., 2021). *P. reticulatum*, another shrub, significantly improves soil quality and nutrient availability, such as nitrogen, phosphorus, and potassium (Fall et al., 2022). It substantially impacts soil hydrology by increasing volumetric water content through enhanced infiltration and raises the rate of nutrient mineralization by promoting beneficial microorganisms, thereby improving soil nutrients. Additionally, it demonstrates very high regeneration abilities. Soils under *P. reticulatum* are characterized by high water availability and provide a favorable environment for microbial activity and diversity that enhance the decomposition of organic matter (Bright et al., 2017). The germination of seeds from these plants is impeded by dormancy due to their hard seed coats, slowing down the germination process and highlighting the need for pretreatment to enhance germination and subsequent seedling growth (Lawan et al., 2021).

Although numerous studies have explored the effects of pregermination treatments on seed germination and seedling survival of *A. nilotica*, *F. albida*, *B. rufescens*, and *P. reticulatum* (Gilani et al., 2019; Barthelemy et al., 2021; Iroko et al., 2021), no methodological consistency regarding various levels of different treatments guarantees a universal generalization of results. Additionally, the effects of different levels of different pregermination treatments on seed germination for many trees native to arid lands are either yet to be, or are meagerly, scientifically tested. Moreover, interpretations of results are predominantly restricted to silvicultural applications and are rarely ecological. Therefore, conducting this study was necessary.

The general aim of this study was to discern the effects of varying durations of seed exposure to acid and hot water, determine the seed germination dynamics of *A. nilotica*, *F. albida*, *B. rufescens*, and *P. reticulatum*, and interpret the results from an ecological perspective for restoration purposes. We hypothesized that the rate of seed viability loss during germination depends on the speed of germination and that this speed is speciesspecific. Understanding seed germination dynamics and survival across different tree species in arid land will enhance our ability to more accurately predict the patterns of natural regeneration of degraded lands. This insight will also assist forest managers in developing innovative management strategies for the more successful restoration and conservation of arid land ecosystems and monitoring and forecasting desertification processes (Dalling et al., 2011; Fox et al., 2012; Mordecai, 2012).

Establishing a large number of tree seedlings, which is essential for afforestation programs, requires knowledge about breaking seed dormancy and achieving synchronous germination. Hence, understanding the seed pregermination treatments that yield better germination results will aid in reforestation projects in arid lands (Malagnoux et al., 2007; Asiedu et al., 2012).

## Materials and methods

### Seed collection

Seeds of four leguminous tree species from arid lands were utilized in this study, including *A. nilotica*, *B. rufescens*, *F. albida*, and *P. reticulatum* (Table 1). The seeds of *A. nilotica*, *F. albida*, and *P. reticulatum* were collected from the wild near the city of Maiduguri, while those of *B. rufescens* were collected near the city of Bauchi; all locations are in North-Eastern Nigeria. Maiduguri is situated at latitude and longitude 11°50′N 13°09′E, with a relief range between 300 and 600 m above sea level. It possesses a semiarid climate, receiving low precipitation (650 mm), below potential evapotranspiration, although not as low as a hyper-arid desert climate. The monthly mean temperatures consistently exceed 20°C, while daily temperatures can soar up to 47°C in April (Jimme et al., 2016). The climate is characterized as a tropical savanna.

**Table 1:** Tree species used in this study

Scientific name	Authority	Family	Common name	Hausa name
*Acacia nilotica*	(L) Willd. ex Del	*Leguminosae*	Egyptian mimosa	Bagaruwa
*Faidherbia albida*	(Del.) A.Chev.	*Fabaceae*	Ana tree	Gawo
*Piliostigma reticulatum*	(DC.) Hochst.	*Caesalpiniaceae*	Camel’s foot	Kalgo
*Bauhinia rufescens*	Lam.	*Leguminosae*	Silver butterfly	Kargon Allah

Conversely, Bauchi City is located at 10.263954°N, 9.811298°, and 616 m above sea level. The hottest months of the year are April and May, with average temperatures ranging from 40 to 56°. In contrast, the coldest months, December and January, have average temperatures of 6.11 and 22°C, respectively (Haruna et al., 2012). The climate, characterized by seasonally alternating wet and dry seasons, experiences rainfall ranging from 600 to 900 mm per year, mostly occurring between May and September (Yusuf and Yusuf, 2008; Concha et al., 2013).

### Seed treatments and experimental design

Seeds were collected from the wild at the end of the rainy season when they were the most abundant. The seeds were collected from at least ten different individual trees per selected species. Seeds from various trees were pooled together, extracted from their pods, and separated from the chaff. Mature seeds – uniform in size, devoid of wrinkles, and showing no signs of damage – were handpicked. Two hundred (200) seeds were counted for each of the acid treatment and the hot water treatment. However, due to scarcity, only 100 seeds were used for the hot water treatment of *A. nilotica* and *F. albida*. The pregermination treatments included soaking in concentrated (100%) sulfuric acid, immersion in boiling water, and a control group (untreated seeds). For the sulfuric acid treatment, the seeds were immersed in the acid within a glass beaker for intervals of 20, 30, 40, 50, and 60 min. The intention behind these prolonged periods of acid treatment was to determine the duration at which the seeds would burn in the acid. The seeds were gently stirred with a glass rod every 5 min and, following the allocated time, were thoroughly rinsed in tap water between 7 to 10 times. Although rinsing the acid-treated seeds five times seemed to remove all the acid, up to 10 times were performed to ensure thorough cleaning. For the boiling water treatment, seeds were encased in an iron mesh and submerged in boiling water for 5, 10, and 15 min (the iron mesh was a window screen net, manufactured by Qinhuangdao Priem Trading Co., Ltd: https://glass-fiberscreen.en.made-in-china. com). Subsequently, the seeds were removed from the boiling water and placed in cool water for 1 min to cool down. Post-treatment, seeds were placed on clinically clean and moist cotton wool inside plastic trays. For the control group, seeds underwent no treatment. The trays were loosely covered with their original lids and placed in a dark cupboard at room temperature for germination (Fredrick et al., 2016; Ameri and Daldoum, 2017). A total of 200 seeds were planted for each treatment. Each treatment was replicated four times; thus, each replicate contained 50 seeds. Similarly, for the batches of 100 seeds, each treatment was replicated four times, with each replicate containing 25 seeds. The seeds were planted following a Randomized Complete Block Design.

### Data collection

For this study, germination was defined as the successful emergence of the radicle tip through the testa (Asiedu et al., 2012). Germinated seeds were counted beginning on the 4^th^ day from the start of the experiment and continued at intervals of every 2 days until the experiment was terminated on the 28^th^ day. After each count, the germinated seeds were removed from the rest of the seeds that had yet to germinate. This removal was performed to avoid recounting the germinated seeds (Ameri and Daldoum, 2017).

### Determination of germination parameters

Data collected was used to calculate different germination parameters and then subjected to Analysis of Variance (ANOVA) followed by Tukey multiple comparison tests (Asiedu et al., 2012). The following germination parameters were estimated:

**Germination energy** was calculated as follows (Willan, 1987):


$$\text{Germination energy}=\frac{\text{Total germinated seeds}}{\text{Number of seeds}}\times 100$$

**Germination period** was regarded as the number of days from the first day of observed germination to the last day when germination ceased (Asiedu et al., 2012).

**Germination capacity** is the number or percentage of seeds to complete germination in a given treatment for the whole experimental period (Dayamba et al, 2014; Fredrick et al., 2016).

**Germination inertia** was calculated as follows:


$$\text{Germination inertia (GI)=}\frac{\text{GE}}{\text{TG}}\times 100$$

where GE is the number of seeds that germinated after the elapse of the experimental period (28 days); and TG is the total number of seeds that germinated in that particular treatment; i.e., the sum of the seeds that germinated during and after the experiment.

**Viability loss** was calculated as follows:


Viability loss=Total number of seeds planted +−Total number of seeds germinated

### Statistical analysis

Analysis of variance (ANOVA), followed by Tukey’s multiple comparison test, was conducted using Minitab^®^ statistical software, version 18.1, to explore possible variations among the treatment means at α = 0.05 level of significance. The results were considered statistically significant at *P* < 0.05 (Dayamba et al., 2014). Germination curves were generated in Microsoft Excel using cumulative data and were used to show the trend of germination throughout the experiments (Chuyong and Acidri, 2015).

## Results

The results of this study showed that pregermination treatments of tested tree seeds (varying durations of exposure to sulfuric acid and boiling water) significantly (α = 0.05) influenced germination dynamics and loss of viability. Seed viability loss was mostly found among seeds with a slower germination speed. However, the level of these effects was dependent on the tree species.

### Effects of pregermination treatments on seeds of Acacia nilotica

**Fig. 1:**
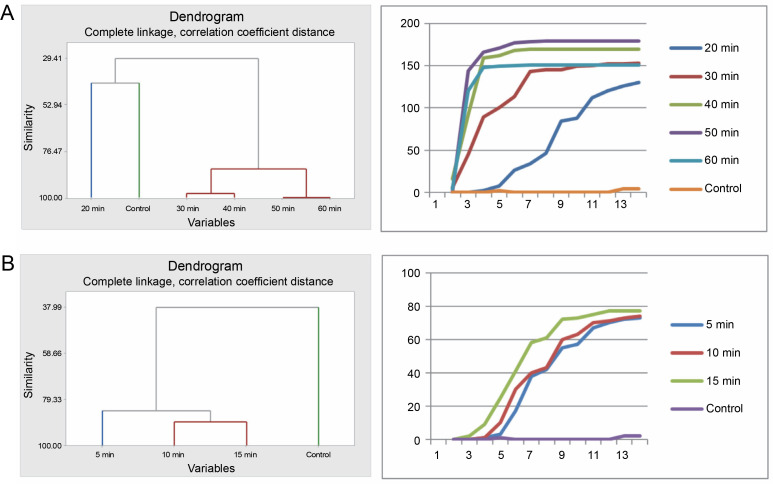
Grouping by similarities from cluster analysis of germination pattern and the trend of germination through the course of the experiment using cumulative data of *A. nilotica* seeds treated with sulfuric acid (A) and hot water (B)

The various pregermination treatments affected the germination patterns of *A. nilotica* seeds in different ways. Cluster analysis revealed that the germination pattern of the control differed from that of the acid-treated seeds. The germination pattern of seeds in the 20-min acid treatment was disparate from that of the remaining acid-treated seeds (30, 40, 50, and 60 min), which exhibited similarity and therefore were categorized into the same group as divulged by the cluster analysis. Correspondingly, the pattern of germination curves, crafted using cumulative data, demonstrates that seeds in the 30, 40, 50, and 60 min treatments followed a similar trend, which diverged from the control and 20 min treatments (Fig. 1A).

**Fig. 2:**
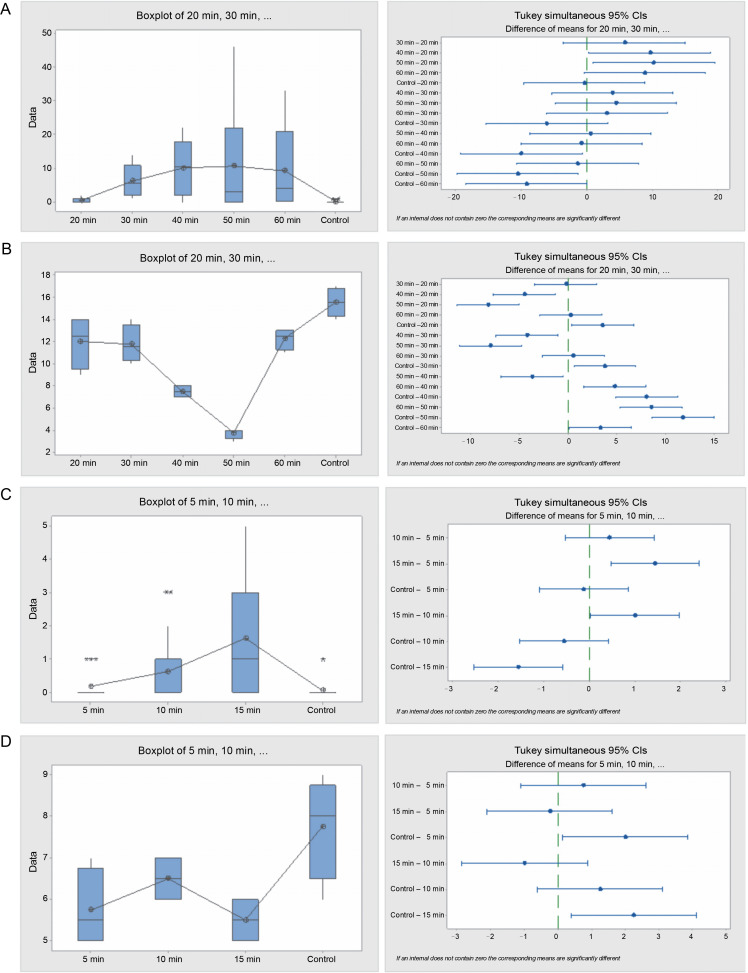
Box plots and their corresponding interval plots of analysis of variance for *Acacia nilotica* seeds germination analyses: germination energy (A) and viability loss (B) for sulfuric acid treatment; germination energy (C) and viability loss (D) for hot water treatment; if an interval does not contain zero, the corresponding means are significantly different

In the hot water treatment, cluster analysis demonstrated that seeds in the 15 and 10 min treatments were similar in their germination patterns or rates. Seeds treated for 5 min germinated at a different rate, as did the seeds in the control. Nevertheless, the graphical curves illustrating the seed germination trend using cumulative data indicate that only the seeds in the control exhibited a significantly different germination pattern, while seeds in the 5, 15, and 10 min treatments followed the same trend (Fig. 1B). Germination energy (the percentage of seeds germinated at day 10) differed significantly (*P* = 0.001) among the seeds treated with sulfuric acid for various durations (Table 2 and Fig. 2).

**Table 2:** Analyses of germination dynamics of *Acacia nilotica* seeds subjected to sulfuric acid and hot water treatment (one-way ANOVA significance level, α = 0.05)

Treatment	Germination energy							Viability loss				
	No. of seeds used	GC [%]	GP	GE [%]	Mean	StDev	*P* value	Total viable seeds	Seeds viability loss [%]	Mean	StDev	*P*-value
Sulfuric acid												
20 min	200	65	> 22	4	0.50 ^b^	0.73	0.001	152 (76)	48 (24)	12.00 ^b^	2.45	0.000
30 min	200	66.5	> 26	50	6.25 ^ab^	4.67		153 (76.5)	47 (23.5)	11.75 ^b^	1.708	
40 min	200	84.5	11	81	10.13 ^a^	8.33		169 (84.5)	31 (15.5)	7.50 ^c^	0.577	
50 min	200	89.5	13	85.5	10.69 ^a^	15.67		185 (95.5)	15 (7.5)	3.75 ^d^	0.500	
60 min	200	75.5	11	74.5	9.31 ^ab^	12.15		151 (75.5)	49 (24.5)	12.25 ^b^	0.957	
Control	200	2	> 26	1	0.13 ^b^	0.34		136 (68)	64 (32)	15.50 ^a^	1.291	
Hot water												
5 min	100	73	> 22	3	0.19 ^b^	0.40	0.000	77	23	5.75 ^b^	0.957	0.016
10 min	100	74	> 22	10	0.63 ^b^	1.09		74	26	6.50 ^ab^	0.577	
15 min	100	77	19	26	1.63 ^a^	1.71		77	23	5.50 ^b^	0.577	
Cont	100	2	17	1	0.06 ^b^	0.25		70	30	7.75 ^a^	1.258	

GP – germination period, GC – germination capacity, GE – germination energy; means that do not share a letter are significantly different

Seeds treated with acid for 40 and 50 min exhibited the highest germination energy of 81 and 85.5% respectively, while seeds in the control and seeds in the 20 min treatment had the least germination energy, of 1 and 4%, respectively. Germination capacity among the acid-treated seeds ranged from 65 to 89.5% for all acid treatments, while that of the control was only 2%. Seeds with longer exposure to acid (40, 50, and 60 min) exhibited the shortest germination period of 11–13 days, while seeds with shorter exposure to acid (20 and 30 min) and those from the control endured the longest germination period, extending beyond the 28 days of the experimental period. Seed viability loss among the acidtreated seeds was also significantly different (*P* = 0.000). The control had the highest seed viability loss of 64 seeds (32% of the total seeds), and the seeds in the 50 min acid treatment had the lowest number of germinated seeds, which was 15 (7.5%).

The hot water treatment on seeds notably influenced the seed germination rate. Seeds subjected to hot water for 15 min exhibited the highest germination energy, which was significantly (*P* = 0.000) higher than that observed in seeds treated for 5 and 10 min, and the control. However, there was no significant difference in the number of germinated seeds between the 5, 10 min treatments, and the control. The germination capacity of the hot water treated seeds lingered between 73 and 77% for all the tree species, while in the control it was meager, at just 2%. The germination period for the seeds from the control and the 15 min treatment were 17 and 19 days, respectively, whereas the seeds from the 5 and 10 min treatments exceeded the 28 days of the experiment. Seed viability loss was significantly (*P* = 0.016) higher in seeds from the control, followed by seeds from the 10-min treatment, but there was no difference between the values in seeds from 5 and 15 min treatments.

### Effects of pregermination treatments on seeds of Piliostigma reticulatum

**Fig. 3:**
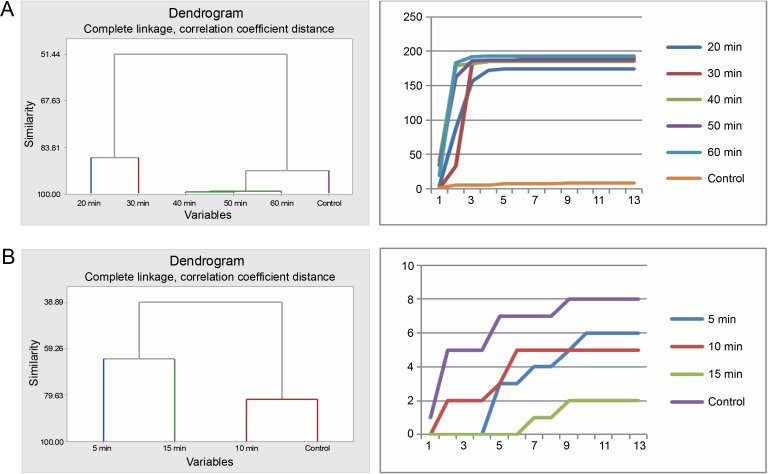
Grouping by similarities from cluster analysis of germination pattern and the trend of germination through the course of the experiment using cumulative data of *Piliostigma reticulatum* seeds treated with sulfuric acid (A) and hot water (B)

The seeds of *P. reticulatum* demonstrated varied germination behaviors depending on the treatment they received. In the sulfuric acid experiment, cluster analysis revealed a similarity in the germination patterns of seeds exposed to longer acid treatments (40, 50, and 60 min) (Fig. 3). Contrastingly, the seeds from the control, 20-min, and 30-min treatments each exhibited distinct germination patterns. The cumulative data-driven germination curve affirmed these observations.

In the hot water treatment, the seeds from the 10-min treatment and the control were grouped together, indicating a similar germination rate. These results diverged significantly from those obtained for the seeds subjected to a 5 min treatment and, in turn, those treated for 15 min, as substantiated by the corresponding germination curves.

**Fig. 4:**
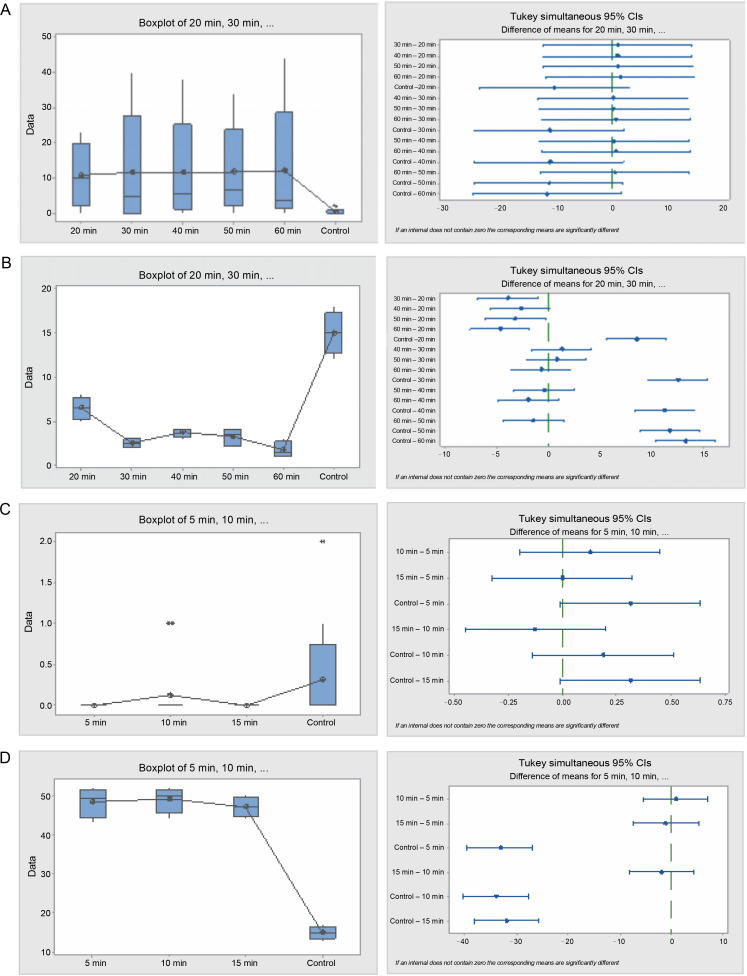
Box plots and their corresponding interval plots of analysis of variance for *Piliostigma reticulatum* seeds germination analyses: germination energy (A) and viability loss (B) for sulfuric acid treatment; germination energy (C) and viability loss (D) for hot water treatment; if an interval does not contain zero, the corresponding means are significantly different

The sulfuric acid experiment displayed no significant difference (*P* = 0.079) among the means of germination energy between seeds treated for varied durations, despite the germination energy of acid-treated seeds ranging from 86 to 96.6%, compared to the mere 2.5% of the control (Table 3 and Fig. 4). Moreover, the germination capacity of acid-treated seeds ranged between 87 and 96.5%, whereas the control lingered at 4% (8 out of the initial 200 seeds planted). However, seed viability loss was significantly different (*P* = 0.000) among seeds from varying treatments and the control — the control exhibited the highest seed viability loss (30%), while the 60-min treated seeds demonstrated the lowest (3.5%). The germination period spanned 7–13 days among the acid-treated seeds, while it stretched to 17 days for the control seeds.

**Table 3:** Analyses of germination dynamics of *Piliostigma reticulatum* seeds subjected to sulfuric acid and hot water treatment (one-way ANOVA significance level, α = 0.05)

Treatment	Germination energy							Viability loss				
	No. of seeds used	GC [%]	GP	GE [%]	Mean	StDev	*P*-value	Total viable seeds	Seeds viability loss	Mean	StDev	*P*-value
Sulfuric acid												
20 min	200	87	9	86	10.75 ^a^	8.85	0.079	174 (87)	26 (13)	6.50 ^b^	1.29	0.000
30 min	200	95	13	92.5	11.56 ^a^	15.75		190 (95)	10 (5)	2.50 ^c^	0.58	
40 min	200	92.5	7	92.5	11.56 ^a^	14.34		185 (92.5)	15 (7.5)	3.75 ^cb^	0.50	
50 min	200	93.5	7	93.5	11.69 ^a^	12.61		187 (93.5)	13 (6.5)	3.25 ^c^	0.96	
60 min	200	96.5	7	96.5	12.06 ^a^	17.27		193 (96.5)	7 (3.5)	1.75 ^c^	0.96	
Control	200	4	17	2.5	0.313 ^a^	0.602		140 (70)	60 (30)	15.00 ^a^	2.45	
Hot water												
5 min	200	3	11	0	0.00 ^a^	0.00	0.042	7 (3.5)	193 (96.5)	48.25 ^a^	3.86	0.000
10 min	200	2.5	9	1	0.13 ^a^	0.34		5 (2.5)	195 (97.5)	49.00 ^a^	3.46	
15 min	200	1	5	0	0.00 ^a^	0.00		12 (6)	188 (94)	47.00 ^a^	2.58	
Control	200	4	17	2.5	0.31 a	0.60		140 (70)	60 (30)	15.00 ^b^	1.633	

GP – germination Period, GC – germination capacity, GE – germination energy; means that do not share a letter are significantly different

In the hot water treatment, no significant difference (*P* = 0.042) was found among seeds from different durations (5, 10, and 15 min) and the control seeds concerning germination energy. Nevertheless, a notable disparity in seed viability loss was evident (*P* = 0.000) between the treatments and the control. The control seeds experienced the lowest viability loss of 30%, whereas the treated seeds encountered a range between 94 and 97%. The germination capacity among seeds from different treatments and the control was merely 1–4% out of the initial 200 seeds planted in each treatment. The germination period lasted longer in the control seeds, peaking at 17 days, and was lowest in the 15 min treatment seeds, at 5 days.

### Effects of pregermination treatments on seeds of Bauhinia rufescens

**Fig. 5:**
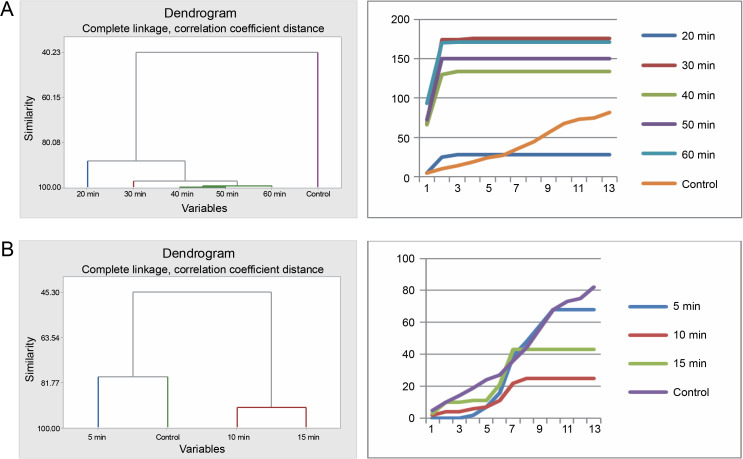
Grouping by similarities from cluster analysis of germination pattern and the trend of germination through the course of the experiment using cumulative data of *Bauhinia rufescens* seeds treated with sulfuric acid (A) and hot water (B)

Germination of *Bauhinia rufescens* seeds differed with different times of exposure to sulfuric acid and hot water treatments. Cluster analysis organized the seeds from the five sulfuric acid treatments (20, 30, 40, 50, and 60 min) and the control into four groups (Fig. 5). Seeds subjected to 40, 50, and 60 min treatments were grouped together, exhibiting similar germination patterns, which significantly differed from the others. Meanwhile, seeds from the 20 to 30 min treatments, and the control each presented distinct germination patterns from one another. Graphical germination curves, demonstrating trends in germination using cumulative data, revealed that seeds in the control and those in the 20-min treatment followed a different trend compared to the rest.

For the hot water treatment results, seeds from the 10 and 15 min treatments displayed similar germination patterns, differing from seeds in the 5-min treatment and the control. However, the control and the 5-min treatment exhibited no significant difference in their germination patterns. This observation was also substantiated by the trends indicated by the corresponding germination curves, derived from the cumulative data.

**Fig. 6:**
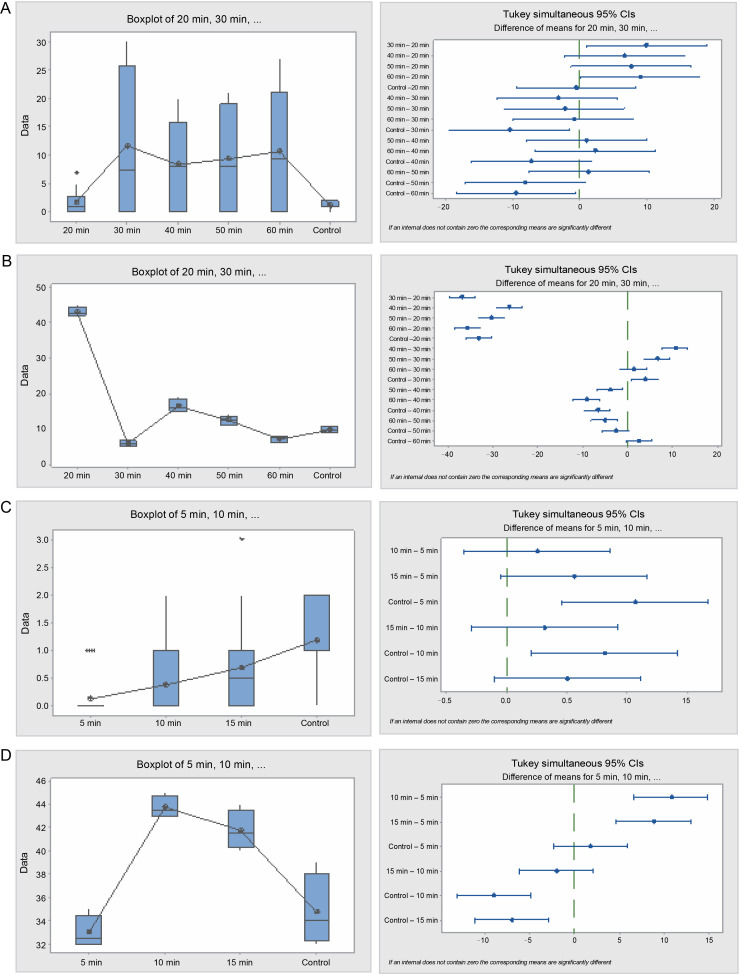
Box plots and their corresponding interval plots of analysis of variance for *Bauhinia rufescens* seeds germination analyses: germination energy (A) and viability loss (B) for sulfuric acid treatment; germination energy (C) and viability loss (D) for hot water treatment; if an interval does not contain zero, the corresponding means are significantly different

Furthermore, seeds treated with sulfuric acid and the control significantly differed (*P* = 0.001) with respect to their germination energy (Table 4 and Fig. 6). Seeds in the 30 and 60-min sulfuric acid treatments yielded the highest seed germination energy, 93 and 85.5% respectively, whereas seeds in the 20 min treatment and the control experienced the lowest germination energy, 14 and 9.5% respectively. Similarly, the germination capacity was highest in the 30 and 60-minute treatments (88 and 85.5%, respectively) and lowest in the 20-min treatment and the control (14 and 41% respectively). The germination period for acid-treated seeds spanned 3–7 days, while for the control, it exceeded 28 days. Seed viability loss was significantly higher in the 20-min treatment seeds at 86%, and lowest in the 30 and 60 min treatment seeds, 12 and 14.5% respectively.

**Table 4:** Analyses of germination dynamics of *Bauhinia rufescens* seeds subjected to sulfuric acid and hot water treatment (one-way ANOVA significance level, α = 0.05)

Treatment	Germination energy							Viability loss				
	No. of seeds used	GC [%]	GP	GE [%]	Mean	StDev	*P*-value	Total viable seeds	Viability loss	Mean	StDev	*P*-value
Sulfuric acid												
20 min	200	14	5	14	1.75 ^b^	2.15	0.001	28 (14)	172 (86)	43.00 ^a^	1.414	0.000
30 min	200	88	7	93	11.63 ^a^	12.65		176 (88)	24 (12)	6.00 ^e^	0.816	
40 min	200	67	5	67	8.38 ^ab^	8.26		134 (67)	66 (33)	16.50 ^b^	1.915	
50 min	200	75	3	75	9.38 ^ab^	9.74		150 (75)	50 (25)	12.50 ^c^	1.291	
60 min	200	85.5	5	85.5	10.69 ^a^	11.09		171 (85.5)	29 (14.5)	7.25 ^de^	0.957	
Control	200	41	> 25	9.5	1.19 ^b^	0.66		161 (80.5)	39 (19.5)	9.75 ^cd^	0.957	
Hot water												
5 min	200	34	13	1	0.13 ^b^	0.34	0.000	68 (34)	132 (66)	33.00 ^b^	1.414	0.000
10 min	200	12.5	15	3	0.38 ^b^	0.62		25 (12.4)	175(87.5)	43.75 ^a^	0.96	
15 min	200	16.5	13	5.5	0.69 ^ab^	0.87		33 (16.5)	167 (83.5)	41.75 ^a^	1.71	
Control	200	41	> 25	9.5	1.19 ^a^	0.66		161 (80.5)	39 (19.5)	34.75 ^b^	3.10	

GP – germination period, GC – germination capacity, GE – germination energy; means that do not share a letter are significantly different

The hot water treatment also influenced the germination pattern of *B. rufescens* seeds. The germination energy was significantly (*P* = 0.000) higher in the 15-min treatment and the control, 5.5 and 9.5% respectively, while seeds in the 5 and 10 min treatments exhibited 1 and 3% respectively. Germination capacity mirrored this pattern; it was higher in the control and 5-min treatments (41 and 34%, respectively) and lower in the 10 and 15 min treatments (12.5 and 16.5%, respectively). Seed viability loss was significantly lower in the control and 5-min treatments and higher in the 10 and 15 min treatments.

### Effects of pregermination treatments on seeds of Faidherbia albida

**Fig. 7:**
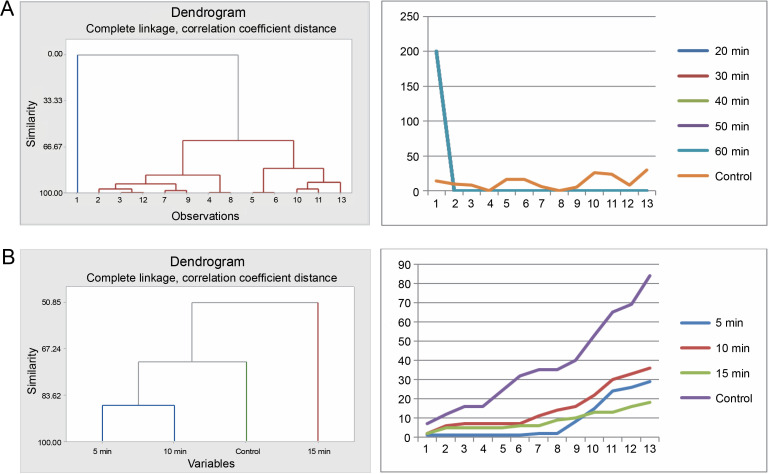
Grouping by similarities from cluster analysis of germination pattern and the trend of germination through the course of the experiment using cumulative data of *Faidherbia albida* seeds treated with sulfuric acid (A) and hot water (B)

The seeds of *F. albida* exhibited a positive response to all durations in the sulfuric acid treatments (20, 30, 40, 50, and 60 min) compared to the control. Cluster analysis indicated that the germination pattern of the control differed from that of the acid-treated seeds, whereas all acid-treated seeds showcased similar patterns. This finding was corroborated using cumulative data of seed germination: all acid-treated seeds followed the same pattern, which was different from the control (Fig. 7A).

**Fig. 8:**
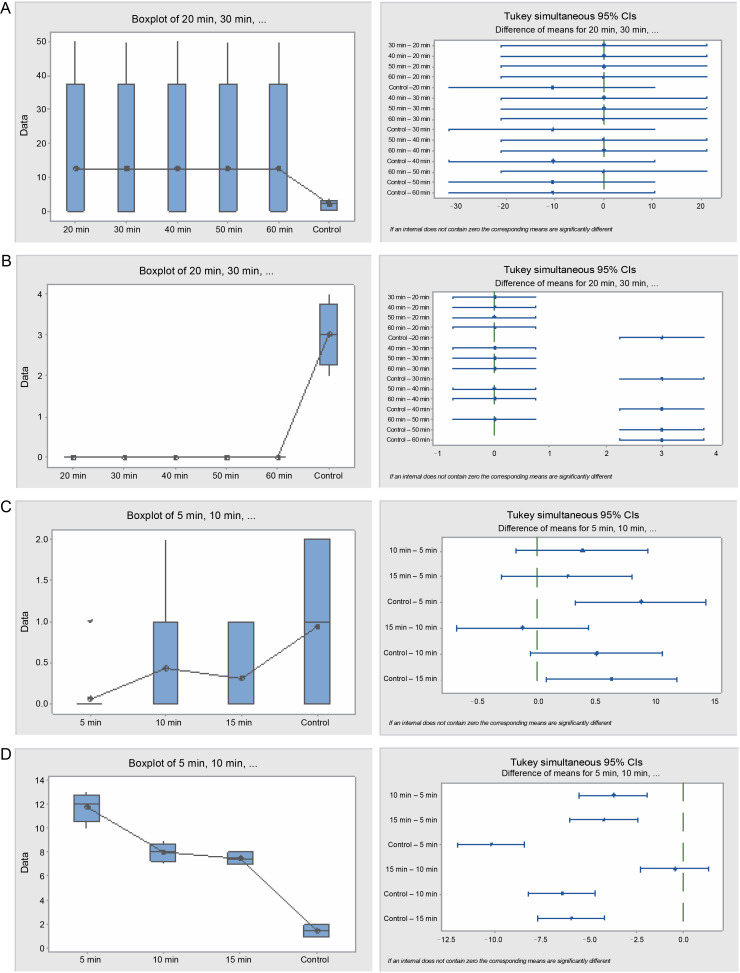
Box plots and their corresponding interval plots of analysis of variance for *Faidherbia albida* seeds germination analyses: germination energy (A) and viability loss (B) for sulfuric acid treatment; germination energy (C) and viability loss (D) for hot water treatment; if an interval does not contain zero, the corresponding means are significantly different

In the hot water treatment, the cluster analysis revealed that seeds subjected to 5 and 10min treatments demonstrated similar germination behavior, which was distinct from the seeds in the 15-min treatment and, subsequently, from the control. These findings are also consistent with the graphical trends of germination using cumulative data (Fig. 7B). No statistically significant difference in germination energy was found between seeds treated with sulfuric acid and those in the control (*P* = 0.621) (Table 5 and Fig. 8). The germination energy of all the acid-treated seeds was 100%, while it was 16% for the seeds in the control.

**Table 5:** Analyses of germination dynamics of *Faidherbia albida* seeds subjected to sulfuric acid and hot water treatment (one-way ANOVA significance level, α = 0.05)

Treatment	Germination energy							Viability loss				
	No. of seeds used	GC [%]	GP	GE [%]	Mean	StDev	*P*-value	Total viable seeds [%]	Seed viability loss [%]	Mean	StDev	*P*-value
Sulfuric acid												
20 min	200	100	1	100	100 ^a^	22.36	0.621	200 (100)	0	0.00 ^b^	0.000	0.000
30 min	200	100	1	100	100 ^a^	22.36		200 (100)	0	0.00 ^b^	0.000	
40 min	200	100	1	100	100 ^a^	22.36		200 (100)	0	0.00 ^b^	0.000	
50 min	200	100	1	100	100 ^a^	22.36		200 (100)	0	0.00 ^b^	0.000	
60 min	200	100	1	100	100 ^a^	22.36		200 (100)	0	0.00 ^b^	0.000	
Control	200	81.5	> 25	16	16 ^a^	1.461		188 (93)	12 (6)	3.00 ^a^	0.816	
Hot water												
5 min	100	14.5	> 25	0.5	0.06 ^b^	0.25	0.001	47	53	11.8 ^a^	1.26	0.000
10 min	100	18	> 25	3.5	0.44 ^ab^	0.63		62	32	8.0 ^b^	0.82	
15 min	100	9	> 25	2	0.31 ^b^	0.48		64	30	7.5 ^b^	0.58	
Control	100	81.5	> 25	6	0.94 ^a^	0.85		94	6	1.5 ^c^	0.58	

GP – germination period, GC – germination capacity, GE – germination energy; means that do not share a letter are significantly different

A significant difference in seed viability loss was observed between the treatments and the control (*P* = 0.000). All the acid-treated seeds germinated within 4 days from the start of the experiment, implying that the germination capacity was 100%, whereas it was 84% for the control. The germination period of the acid-treated seeds for all durations was merely 2 days, while the germination period of the control seeds extended beyond the 28 days when the experiment was terminated.

In the hot water treatment, germination energy varied significantly (*P* = 0.000) between the control and treatments (5, 10, and 15 min). The control exhibited the highest germination energy (6%), followed by the 10-min treatment (3.5%). Germination capacity was also higher in the control (42%), followed by the 10-min treatment (18%), and was lowest in the 15-min treatment (9%). Seed viability loss was significantly (*P* = 0.000) varied among the hot water treatment seeds. Viability loss was greater in the 5-min treatment than in the 10 and 15 min treatments, which, in turn, were greater than the control.

### Germination inertia and viability loss for the untreated seeds of A. nilotica, B. rufescens F. albida, and P. reticulatum

**Fig. 9:**
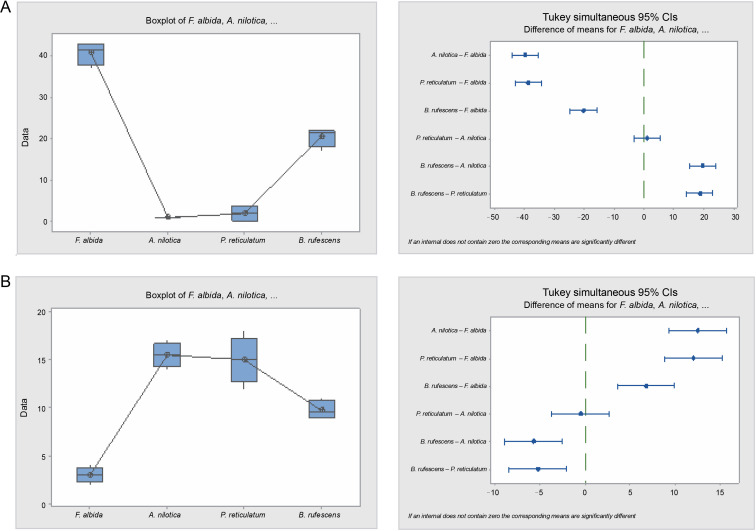
Box plots and their corresponding interval plots of analysis of variance analyses of germination inertia (A) and viability loss (B) for *F. albida, A. nilotica, P. reticulatum* and *B. rufescens* seeds; if an interval does not contain zero, the corresponding means are significantly different

Data obtained for the seeds that did not undergo any treatment were also analyzed for germination inertia and viability loss. Germination inertia, measuring the delay in the germination process of viable seeds, revealed that *A. nilotica* and *P. reticulatum* exhibited the highest germination inertia at 98.5 and 94.3%, respectively (Table 6 and Fig. 9). This means that approximately 94–99% of their viable seeds failed to germinate during the 28 days of the experiment. Conversely, *B. rufescens* and *F. albida* demonstrated the lowest germination inertia, recording 49.1 and 13.3%, respectively. Analyses of their germination capacity (the total seeds that germinated during the 28-day experiment period) suggested that these differences were statistically significant (*P* = 0.000), except for the difference between *A. nilotica* and *P. reticulatum*. The seed germination capacities for the control seeds were as follows: 2% for *A. nilotica*, 82% for *B. rufescens*, 84% for *F. albida*, and 8% for *P. reticulatum*.

**Table 6:** Analyses of germination inertia and viability loss for *F. albida, A. nilotica, P. reticulatum* and *B. rufescens* seeds using the control data (one-way ANOVA at significance level, α = 0.05)

Analyses of germination inertia							Analyses of viability loss			
Tree	Viability [%]	* GI [%]	** GC [%]	*** Mean	StDev	P-value	Viability loss [%]	*** Mean	StDev	P-value
*F. albida*	93	13.3	81.5	40.75 ^a^	2.87	0.000	6	15.50 ^c^	0.816	0.000
*A. nilotica*	68	98.5	2	1.00 ^c^	0.00		32	15.00 ^a^	1.291	
*P. reticulatum*	70	94.3	4	2.00 ^c^	1.83		30	9.75 ^a^	2.45	
*B. rufescens*	80.5	49.1	41	20.50 ^b^	2.38		19.5	3.00 ^b^	0.957	

* GI – germination inertia,

** GC – germination capacity;

*** means that do not share a letter are significantly different

Results from the analyses of viability loss mirrored those of the germination inertia proportionately. The viability loss for *A. nilotica* and *P. reticulatum* was higher (32 and 30%, respectively) compared to *B. rufescens* and *F. albida*, which were 19.5 and 6%, respectively. Moreover, these differences were statistically significant (*P* = 0.000), except between *A. nilotica* and *P. reticulatum*.

## Discussion

Trees of arid lands – ecologically challenging environments characterized by climatic unpredictability and highly seasonal variability – have evolved seed dormancy, granting them a level of resistance to germination under conditions that would otherwise promote germination in nondormant seeds (Willis et al., 2014; Kildisheva et al., 2020). One prevalent strategy for inducing seed dormancy, especially within *Acacia* spp., involves the development of a thick, water-impermeable seed coat (Iroko et al., 2021). The degree of dormancy can exhibit significant variability among seeds of different tree species or even among seeds of the same species from different geographic locations (Fredrick et al., 2016). Numerous studies have demonstrated that pregermination treatments can significantly lead to quicker and uniform seed germination by increasing the permeability of the seed coat to water. Acid scarification and hot water treatments are commonly utilized pregermination strategies (Tadros et al., 2011; Adewole et al., 2017). Acid scarification and immersing seeds in hot water for a specific period break, disrupt, or degrade the seed coat structure, thereby enhancing the imbibition of water and oxygen uptake by the seeds (Naim et al., 2015). The results of this study indicate that pregermination treatments of seeds, involving varying exposure times to sulfuric acid or boiling water, significantly impacted seed germination dynamics and viability loss in the tested tree species (*A. nilotica*, *B. rufescens*, *F. albida*, and *P. reticulatum*). Nevertheless, the level of these effects depends on the tree species, as previously noted by Fredrick et al. (2016).

### Effects of pregermination treatments with sulfuric acid on seed germination dynamics

#### Germination energy

Germination energy, defined as the percentage of seeds germinated by day 10 (Genes and Nyomora, 2018), significantly differs among the seeds of different species and treatments with different levels of exposure to sulfuric acid. In this study, generally, longer times of exposure to acid gave better germination, characterized by more even and faster (Sardoei et al., 2014). This concurs with earlier findings, suggesting that the hard external seed coat is responsible for dormancy because a longer time of exposure to acid resulted in greater degradation of the seed coat (Naim et al., 2015). However, the sensitivity of the seeds to acid exposure exhibited variances among the different plant species under investigation. At shorter times of exposure, the acid treatment had no effect germination of *A. nilotica*, a detrimental effect on *B. rufescens*, and a positive effect on *P. reticulatum* and *F. albida*. Previous research indicates that *A. nilotica* seeds possess a thicker seed coat compared to most *Acacia* species (Warrag and Eltigani, 2005). Paradoxically, the seeds of *A. nilotica* were more sensitive to the acid treatment at longer times of exposure Specifically, germination energy was notably diminished in *A. nilotica*, while it was either enhanced or un-affected in *B. rufescens*, *F. albida*, and *P. reticulatum*. The best acid treatment that improved the germination energy of the four tested tree species was as follows: 50 min for *A. nilotica*; 30 min for *B. rufescens* ; 20 min for *F. albida* ; and 60 min for *P. reticulatum*. These findings align with those of previous studies; Asiedu et al. (2012) and Diallo et al. (1996) reported that *B. rufescens* germinated most effectively after 30 min of treatment with concentrated sulfuric acid, while Opoku et al. (2018) found that a 45 min soak in concentrated sulfuric acid yielded optimal germination results for *B. rufescens*. Additionally, Yousif et al. (2020) noted that a 60-min sulfuric acid pretreatment was most suitable for *A. nilotica*.

#### Seed viability loss

In this study, seed viability loss was defined as the total number of untreated seeds that both failed to germinate and decayed by the end of the experiment. Variability in the loss of seed viability was observed among different species and treatments. Generally, a significantly higher loss of viability was identified in treatments resulting in lower germination energy of seeds; that is, higher germination energy correlated with lower viability loss. Furthermore, for *F. albida*, Fredrick et al. (2016) reported that pregermination treatments exerted no significant effects on the final germination percentage, even over extended periods. However, the present study showed that the viability of untreated seeds was significantly affected during the experimental period.

#### Seed germination capacity

In this study, the germination capacity is defined as the total percentage of seeds that germinated from the first day to the last day of the experiment (28 days). The results revealed that germination capacity was akin to germination energy, i.e., the longer the period of exposure to acid, the greater the germination capacity was for all species. The plausible explanation for why germination capacity followed a similar pattern to germination energy is that most of the seeds germinated within the first few days from the start of the experiment. According to Fredrick et al. (2016), treating seeds of *F. albida* with concentrated sulfuric acid for 30 min yielded a 90% germination capacity. In contrast, this study found that treating seeds of *F. albida* with concentrated sulfuric acid for 30 minutes resulted in a 100% germination capacity. Dayamba et al. (2014) reported that treating *P. reticulatum* with sulfuric acid for 30 min had no significant effects, a finding that markedly differs from the results of this study. Here, a 30-min treatment of *P. reticulatum* with sulfuric acid yielded a 95% germination capacity, in stark contrast to the 4% observed for the control, a significantly different outcome. However, for *A. nilotica*, Nasr et al. (2013) reported that a sulfuric acid treatment for 45 min facilitated about 80% germination. This result aligns closely with the findings of this study, where 84–90% germination of *A. nilotica* seeds was observed.

#### Germination period

The germination period was defined as the number of days from the initial day of germination to the final day when no additional seeds germinated. Seeds that underwent acid treatment for extended exposure periods exhibited the highest germination energy and capacity, as well as the shortest germination period. Most seeds were subjected to shorter exposure periods and those from the control failed to germinate throughout the experiment, which lasted 28 days. An exception was found in the case of *F. albida*, which exhibited the shortest germination period among all treated seeds. Since the seed coat of *F. albida* is hard, more time is required for seeds to germinate in nursery establishment, and the percentage of seed germination is low (Fredrick et al., 2016).

### Effects of pregermination treatments with hot water on seeds of some leguminous trees

The germination patterns of seeds from different tree species were distinctly influenced by hot water treatments of varying durations. Germination of *A. nilotica* significantly improved with hot water treatments, a finding previously noted by Gilani et al. (2019), though contradicted by Satti et al. (2016). The germination capacity of *A. nilotica* was 73–77%, compared to 2% of the control, corroborated by results obtained by Nasr et al. (2013). This study unveiled that longer exposure to hot water enhanced the germination of *A. nilotica* seeds. Consequently, hot water treatment could feasibly replace sulfuric acid in the pre-germination treatment of *A. nilotica* seeds. However, acid treatment provided a more substantial improvement in breaking the seed dormancy of this species. The hot water treatment also resulted in comparatively lesser seed viability loss. Conversely, acid treatment led to higher germination energy (a higher rate of germination) than the hot water treatment.

Moreover, this study disclosed that hot water treatment adversely affected the seeds of the remaining species tested (*B. rufescens*, *F. albida*, and *P. reticulatum*) compared to the control. Seed viability loss among these species ranged from 53 to 96%. This result concurs with reports from Fredrick et al. (2016), who utilized 80°C hot water and allowed the seeds to cool in the water for 24 h, and Diallo et al. (1996), who used hot water treatment for 10, 80, or 300 s. Both studies revealed that hot water exerted negative or no effects on the germination of *F. albida* seeds. Conversely, according to reports by Asiedu et al. (2011), who treated seeds of *B. rufescens* for 10 s in boiling water, and Opoku et al. (2018), who treated seeds of *B. rufescens* for 60 min in 65°C water, such hot water treatments yielded superior germination results than the control in *B. rufescens*. Higher temperatures have been previously reported to negatively impact the germination of acacia seeds (Chuyong and Acidri, 2015). Prolonged contact of seeds with hot water leads to the inactivation or destruction of enzymes crucial for initiating germination and, ultimately, the death of the seed embryo (Chuyong and Acidri, 2015; Fredrick et al., 2016; Amerin and Daldoum, 2017). However, this study’s results demonstrate that the effect of prolonged seed incubation at high temperatures is contingent upon the tree species (Tchatchoua et al., 2019).

#### Seeds germination inertia and viability loss for the controls of A. nilotica, B. rufescens F. albida, and P. reticulatum

An important parameter providing insights into the ecological dimensions of seed germination is the resistance of viable seeds to initiate germination (Willis et al., 2014). This, essentially, pertains to the degree or an estimate of a viable seed’s resistance to germinate even when conditions are optimal for germination. For simplicity, in this study, it is termed “germination inertia.” It shows a slowness in the germination process. This is the opposite of germination energy or germination capacity; that is, the higher the germination energy or capacity, the lower the germination inertia. This aspect elucidates how various tree species spread their germination batches over a period of time to avoid chances of death, hence showcasing their adaptation to, or their ability to endure, arid land conditions (Willis et al., 2014). The findings from this study revealed that *A. nilotica* and *P. reticulatum* exhibited the highest germination inertia. Approximately 94–99% of their un-treated seeds failed to germinate during the experiment (28 days). However, postacid treatment, they all germinated, indicating they remained viable until the experiment concluded. *F. albida* and *B. rufescens* demonstrated the lowest germination inertia, i.e., their seeds germinated both earlier and faster than those of the other tested species.

Loss of seed viability is also known to occur in the presence of moisture, which is enough to initiate the complete germination process (De Vitis et al., 2020; Mbi et al., 2022). Slight scarification of the seed coat may permit marginal water imbibition, succeeded by a slower germination process (Ibrahim and Hawramee, 2019). This provides an opportunity for rapid-growing saprophytic or pathogenic fungi to attack the softened seed coat, swiftly infiltrate the endosperm and the embryo, thereby inducing seed decay. Dalling et al. (2011) reported that viable seeds can harbor pathogenic fungi on their surface or within their internal tissues. When the germination process is swift, the probability of infection by the pathogen can be minimized. However, when the germination rate is leisurely, the seeds are typically terminated by the pathogens (Dalling et al., 2011). In their natural environment, the partial breaking of seed dormancy may conceivably be induced by the fungi themselves, as fungi are known to facilitate the breaking of dormancy by assaulting and degrading the seed coat (Coronado et al., 2011; Mordecai, 2012). Additionally, the degree of dormancy can fluctuate among seeds from different tree species, different individuals of the same tree species, or individual seeds from the same tree (Wagmann et al., 2012; Liyanage and Ooi, 2015; Kaye et al., 2018; Wyse et al., 2018; Klupczynska and Pawłowski, 2021). Furthermore, seed microhabitats, which influence environmental variables – specifically temperature and moisture availability – vary spatially (Dalling et al., 2011; Opoku et al., 2018). Thus, the rate at which fungi degrade seed coats may be slowed due to sub-optimal environmental conditions, the composition of the fungal species involved, or the degree of the hardness of the seed coat. Additionally, the seed coat degradation level by fungi is influenced by the involved fungal species and prevailing environmental conditions. Numerous abiotic factors are known to impact the outcomes of interactions among microorganisms, which may either be mutualistic or antagonistic toward one another (Hiscox et al., 2018). These factors undeniably afford the seed varying germination rates and sub-sequent viability loss.

## Conclusions

The germination treatments o seeds from *A. nilotica*, *B. rufescens*, *F. albida*, and *P. reticulatum* have been significantly influenced by pregermination treatments, involving varying durations of exposure to sulfuric acid and boiling water. Acid treatments led to better seed germination than hot water treatments except for *A. nilotica* where the hot water treatment performed pretty well. Moreover, extended durations of acid exposure typically yielded enhanced germination energies across the seeds. Optimal acid treatment durations for improved germination across the studied tree species were identified as follows: 50 min for *A. nilotica*; 30 min for *B. rufescens*; 20 min for *F. albida*; and 60 min for *P. reticulatum*. *A. nilotica* and *P. reticulatum* demonstrated higher germination inertia, whereas *F. albida* and *B. rufescens* exhibited lower values. Hot water treatment had negative effects on the seed germination of *B. rufescens*, *F. albida*, and *P. reticulatum*, contributing to significant viability loss.

The newfound insights into seed germination dynamics, especially pertaining to germination inertia and viability loss, will equip forest managers with the capability to accurately predict seed survival probabilities. This, in turn, ensures the successful implementation of forest regeneration programs in arid lands. Furthermore, understanding the seeds’ responses of these plant species to hot water and acid treatments will prove beneficial in plantation programs, where a large number of simultaneously germinating seeds are required for seedling nurseries.

## Declaration

We declare that this manuscript has not been previously published and is not under consideration in the same or sub-stantially similar form in any other peer-reviewed media.
